# Cutaneous diphtheria in a child returning from visiting friends & relatives in Burkina Faso: a case report

**DOI:** 10.1007/s15010-025-02636-3

**Published:** 2025-09-25

**Authors:** G. Pagani, M. Ticozzelli, F. Bassani, S. Sciarrabba, R. Agistri, L. Pogliani, B. Osnaghi, C. Pavia, L. Vismara, S. Rusconi

**Affiliations:** 1 UOC Malattie Infettive, ASST Ovest Milanese, Ospedale Nuovo di Legnano, Legnano, MI Italy; 2 Università degli Studi di Milano, DIBIC, Milano, Italy; 3 UOC Pediatria e Neonatologia, Ospedale Nuovo di Legnano, ASST Ovest Milanese, Legnano, MI Italy; 4 UOC Microbiologia, Ospedale Nuovo di Legnano, ASST Ovest Milanese, Legnano, MI Italy

**Keywords:** Diphtheria, Cutaneous, Emerging, Epidemiology

## Abstract

We present a case report of an 8-year-old African origin, Italian born girl, fully immunized for Diphtheria, with multiple ulcers caused by *Corynebacterium diphtheria*. The clinical event was preceded by a recent trip to Burkina Faso in a rural area. The atypical presentation with ulcers spread to the entire body and particularly to one leg, led to a bacterial swab, from whose culture the pathogen emerged. The girl was initially empirically treated with amoxicillin/clavulanate and then azithromycin. Cutaneous forms are the most common of all forms and have a relevant spreading role. This case is of particular concern in the context of a rising number of cases in Europe, and it’s peculiar since it doesn’t regard immigrants and refugees, whereas it regards a fully immunized child who traveled from an endemical country.

## Case report

An 8-year-old girl born in Italy but of African origin was brought to the Emergency Department for multiple non-healing ulcers spread on the entire body and, in particular, on both legs and the face.

The child, being born in Italy, had received complete vaccinations according to the Italian vaccine program: dTP, HiB, HBV, poliomyelitis, measles, rubella, and mumps; her past medical history was unremarkable.

She had returned 7 days before from Burkina Faso (their mother’s Country of Origin), where they stayed for approximately one month (from August 1 to September 2) in a rural area to visit friends and relatives (VFR).

During their stay, she didn’t have any contact with sick people, nor did she have any systemic symptoms, except for an episode of self-limiting diarrhea.

Her mother reported abundant precipitations and that the children in the village played multiple times in flooded streets. She also reported numerous insect bites.

Skin lesions began to appear towards the end of her stay and progressively worsened during the week after their return, prompting their mother to bring them to the ED.

There, she was evaluated by both a pediatrician and an infectious disease specialist.

**Fig. 1:**
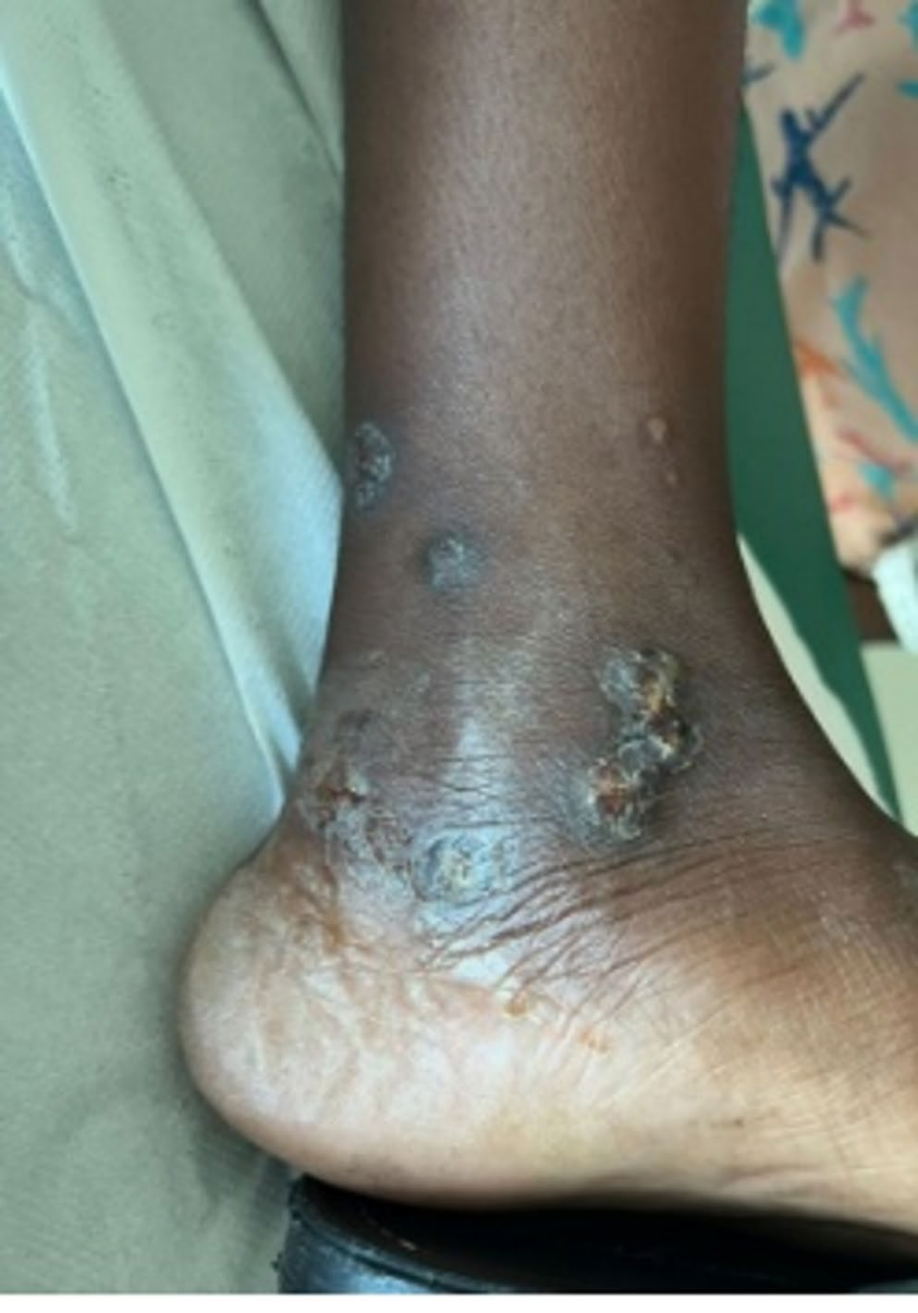
Ulcers at presentation (foot)

**Fig. 2:**
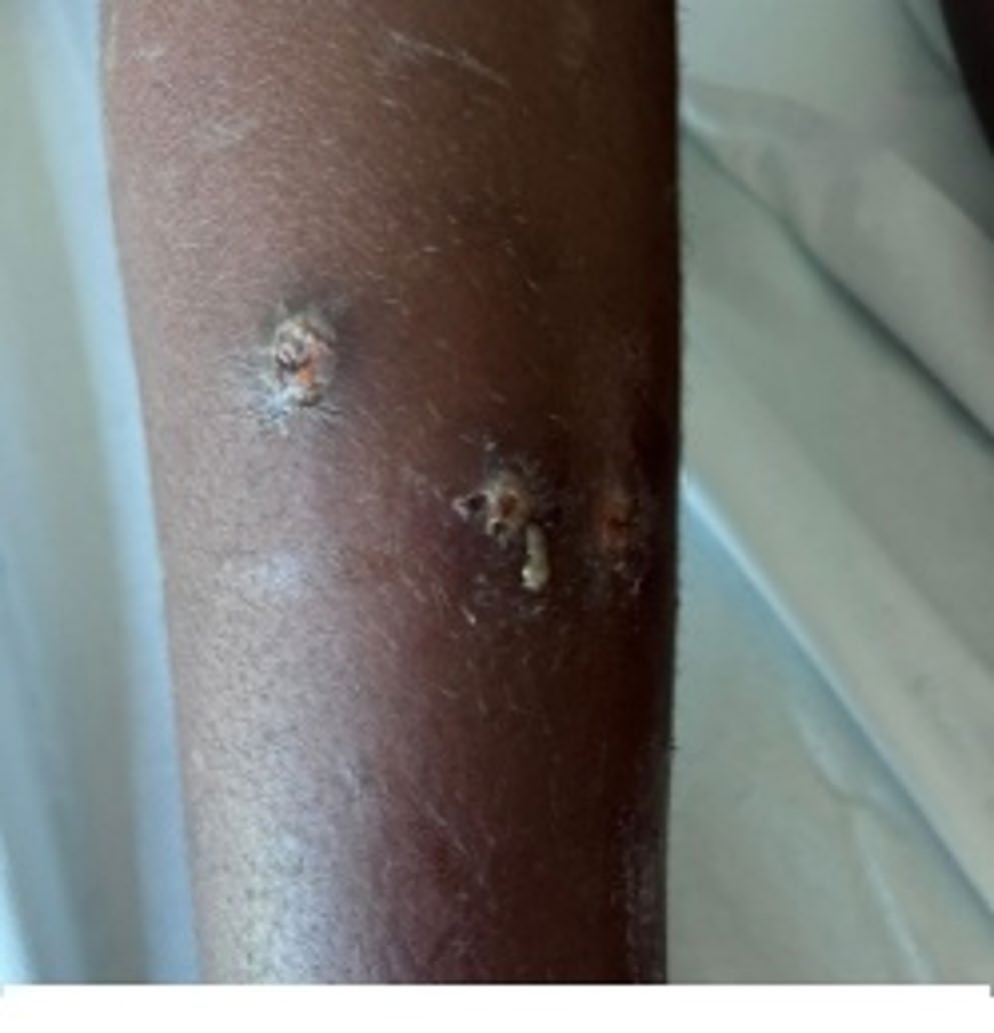
Ulcers at presentation (leg)

She presented numerous bilateral well-demarcated shallow skin ulcers spread all over her body, which appeared during their trip and progressively worsened over 15 days. Purulent discharge was not present at the time of the visit, and the ulcers had a whiteish dry wound bed (Figs. 1 and 2). The lesions were especially present on both legs, arms, and face. Genitals, palmoplantar areas, and mucosal tissues were spared.

She did not report any systemic symptoms; in particular, they did not report malaise, fever, cough, or sore throat. At the time of the presentation, they were in good health and did not report any other complaints except for intense itching.

Due to the atypical presentation of the ulcers, a bacterial swab and blood tests were performed.

Blood tests were unremarkable, except for a slight elevation in Eosinophils count and a slight elevation of CRP.

She was then discharged with empiric antibiotic therapy as for superinfected arthropod bites (amoxicillin/clavulanate [AMC] 100 mg/kg divided into three doses q8h).

Three days later, culture from the lesions’ swab came back positive for both *Corynebacterium diphtheriae*. The isolate was susceptible to linezolid, rifampin, and vancomycin, resistant to tetracycline and clindamycin, and had intermediate susceptibility to penicillin and ciprofloxacin. Additionally, growth of rare colonies of *Streptococcus pyogenes* was present, which was interpreted as either a contaminant or bacterial super-infection.

After identification of the causative agent, a follow-up visit on September 16th (7 days after presentation to our ED) was scheduled: the cutaneous lesions had already drastically improved in both number and size, and the child was well, without any systemic symptom. Antimicrobial therapy was modified by adding azithromycin (500 mg QD for 7 days), according to international guidelines), and was given to all household members as a prophylactic measure [1].

All household members were screened with throat swabs and prophylactically treated with a course of azithromycin.

A surveillance report was sent to Italian Health Authorities (Istituto Superiore di Sanità, ISS), together with a sample of the isolate, to confirm both identification and the presence of the diphtheritic toxin, according to Italian laws.

Identification as *C. diphtheriae* subtype mitis was performed by molecular analyses by ISS. There was no evidence of toxin production.

Another follow-up visit was scheduled a week later. No new skin lesions appeared, and the existing ones had evolved into scars with no signs of active infection.

Antimicrobial therapy was stopped after a total of 14 days of AMC and 10 days of azithromycin, no adverse effects were reported.

## Materials and methods

The sample of cutaneous lesions was collected in Copan eSwab^®^ for aerobic, anaerobic fastidious bacteria (eSwab^®^, Copan, Italy) and inoculated, using the modular open-platform WASP,​ on 5% sheep blood agar, chocolate agar, and MacConkey agar and incubated aerobically at 37 °C. After 10 to 12 h of incubation, minute grayish circular low-convex hemolytic colonies were seen on 5% sheep blood agar; Gram staining from the colonies grown on 5% sheep blood agar showed short gram-positive bacilli in cuneiform arrangement with swelling on one or both ends (club-shaped). In the same sample, we also observed β-hemolytic colonies growth on sheep blood agar; consequently, we proceeded with the isolation of the two strains to obtain separate colonies. Identification of the microorganisms was obtained using matrix-assisted laser desorption ionization-time of flight mass spectrometry (VITEK^®^ MS PRIME MALDI-TOF). *C. diphtheriae* and *Streptococcus pyogenes* were identified with a confidence interval of 99%. The antimicrobial susceptibility was tested with Card ST03 VITEK^®^ 2 AST (bioMerieux, Marcy l’Etoile, France) for *Streptococcus pyogenes* and by disk diffusion method of Kirby-Bauer for *C. diphtheriae*. Antimicrobial susceptibility testing results were interpreted using clinical breakpoints available in the European Committee on Antimicrobial Susceptibility Testing (EUCAST) breakpoint tables for interpretation of MICs and zone diameters Version 14.0, valid from 2024-01-01.

At the National Reference Laboratory for Diphtheria at ISS, species identification and the presence of diphtheria tox gene were verified using Real-Time Polymerase Chain Reaction. The biovar of the isolate was determined using API Coryne Test System (BioMérieux, Marcy L’Etoile, France) [2].

## Discussion

### Clincal presentation

Cutaneous diphtheria is a form of diphtheria caused both by toxigenic and non-toxigenic strains of *C. diphtheriae* and occasionally by other species of Corynebacterium such as *C. ulcerans* and *C. pseudotuberculosis*,* C. belfantii*,* C. rouxii*,* C. silvaticum*,* and C. ramonii*, collectively named *Corynebacteria* of the *diphtheria* species complex (CdSC) [3].

Although mostly known for its “classic” systemic presentation, characterized by pseudo-membranous pharyngitis, croup, and systemic involvement, this form represents only 10–30% of diagnosed diphtheria cases. Cutaneous form represents, however, the majority of cases (57–86%) of diphtheria cases and has an important role in the spread and transmission of the disease [4, 5].

### Epidemiology

Since the widespread adoption of universal vaccination, the disease has become rare in high-income countries (HIC), especially in its systemic form, while it remains widespread in low-to-middle-income countries (LMIC), especially in Sub-Saharan Africa, South America, Central Asia, and South-East Asia, with recent outbreaks in Venezuela, Haiti, Indonesia, Yemen, and Bangladesh (among the Rohingya refugees from Myanmar) [3].

However, due to the necessity of repeated doses during a lifetime (one every ten years, according to WHO recommendations), waning immunity has been described in HIC, especially in older age groups [6, 7].

Recently, a remarkable surge of diphtheria cases, particularly the cutaneous form, has been described in Central and Northern Europe, mainly tied to refugees coming from endemic areas. In particular, the European Centre for Disease and Control (eCDC) reported 92 cases of diphtheria in Europe from January 1 to September 26, 2022, of which 63 were caused by toxigenic strains. The majority of cases were reported from Austria, France, Belgium, and Germany; by contrast, only 1 case was reported from Italy in 2022, and only 4 cases were reported during the 2018–2022 period [8].

To our knowledge, this is the second case report in literature about cutaneous diphtheria in Italy: in 2023, Taccari et al. described a refugee from Bangladesh who probably acquired the infection during his stay in a detention camp in Libya [9].

The clinical presentation of our case is consistent in clinical characteristics with both the aforementioned Italian case report and case series from Germany and France: the majority of cases reported multiple painful and/or itching lesions, more numerous on the lower extremities, mainly ulcerated and with a fibrinous bottom, frequently co-infected with other cosmopolitan bacteria (mostly *S. aureus* and *S. pyogenes*) [5, 9, 10].

Epidemiological characteristics of our patient are, however, very different from the majority of cases recently reported in Europe, which were in refugees or migrants who had spent time in overcrowded and hygienically poor conditions during their travel. They were mostly young men who frequently presented other health issues or were generally unfit due to the migration. The area of acquisition was Central or South Asia or North Africa in the majority of cases [5, 10, 11]. Their demographical and epidemiological characteristics are, however, probably due to a selection bias, as they represent the majority of refugees more than the typical patient with diphtheria [12].

In our case, on the contrary, a young, healthy child born in Italy was infected during a trip to visit friends and relatives in Sub-Saharan Africa. This is more similar to the cases reported in the UK, a country relatively less impacted by recent refugee flows and characterized by a multi-ethnic population and a high frequency of VFRs [13].

### Role of immunization

In this case, the patient was fully immunized for diphtheria, as per the Italian Vaccination program, according to which a full course of immunization against diphtheria, tetanus, and pertussis (dTP) is compulsory for all children. Vaccination immunizes against the diphtheric toxin and prevents toxin-related complications [3]. However, it does not prevent colonization or cutaneous infection, as shown in the UK study, where all cutaneous cases were either fully or partially vaccinated [14].

It is important to notice that vaccination coverage for diphtheria in Italy is almost universal in children (> 95% until 36 months of age). Still, a recent study estimated a drastically lower vaccine coverage rate (VCR) in the 19-to-50-years-old age group, with some regions with an estimated VCR lower than 10% [15], leaving ample segments of the population potentially susceptible to the infection.

## Conclusion

Diphtheria is still a rare disease in Europe, but its incidence has been on the rise in recent years, mainly due to migratory movements either from or through endemic countries.

Our case report highlights the importance of raising awareness among travelers from endemic regions, where the diagnosis of cutaneous diphtheria must be considered in chronic cutaneous lesions. Although more common in refugees, this diagnosis must also be considered in travelers in endemic areas, particularly in those reporting longer stays such as VFRs.

Awareness, early recognition and reporting to health authorities are essential to avoid the risk of re-importation of the disease in our country, characterized by waning immunity and suboptimal vaccine coverage in adults.

## Data Availability

No datasets were generated or analysed during the current study.
